# Assessing the Relationship between Lung Density and Function with Oxygen-Enhanced Magnetic Resonance Imaging in a Mouse Model of Emphysema

**DOI:** 10.1371/journal.pone.0151211

**Published:** 2016-03-15

**Authors:** Magdalena Zurek, Louise Sladen, Edvin Johansson, Marita Olsson, Sonya Jackson, Hui Zhang, Gaell Mayer, Paul D. Hockings

**Affiliations:** 1 Personalised Healthcare and Biomarkers, Innovative Medicines and Early Development Biotech Unit, AstraZeneca, Gothenburg, Sweden; 2 Respiratory, Inflammation & Autoimmunity, Innovative Medicines and Early Development Biotech Unit, AstraZeneca, Gothenburg, Sweden; 3 Discovery Sciences, Innovative Medicines and Early Development Biotech Unit, AstraZeneca, Gothenburg, Sweden; 4 Drug Safety and Metabolism, Innovative Medicines and Early Development Biotech Unit, AstraZeneca, Gothenburg, Sweden; 5 MedTech West, Chalmers University of Technology, Gothenburg, Sweden; Research Center Borstel, GERMANY

## Abstract

**Purpose:**

A magnetic resonance imaging method is presented that allows for the simultaneous assessment of oxygen delivery, oxygen uptake, and parenchymal density. The technique is applied to a mouse model of porcine pancreatic elastase (PPE) induced lung emphysema in order to investigate how structural changes affect lung function.

**Method:**

Nine-week-old female C57BL6 mice were instilled with saline or PPE at days 0 and 7. At day 19, oxygen delivery, oxygen uptake, and lung density were quantified from T1 and proton-density measurements obtained via oxygen-enhanced magnetic resonance imaging (OE-MRI) using an ultrashort echo-time imaging sequence. Subsequently, the lungs were sectioned for histological observation. Blood-gas analyses and pulmonary functional tests via FlexiVent were performed in separate cohorts.

**Principal Findings:**

PPE-challenged mice had reduced density when assessed via MRI, consistent with the parenchyma loss observed in the histology sections, and an increased lung compliance was detected via FlexiVent. The oxygenation levels, as assessed via the blood-gas analysis, showed no difference between PPE-challenged animals and control. This finding was mirrored in the global MRI assessments of oxygen delivery and uptake, where the changes in relaxation time indices were matched between the groups. The heterogeneity of the same parameters however, were increased in PPE-challenged animals. When the oxygenation status was investigated in regions of varying density, a reduced oxygen-uptake was found in low-density regions of PPE-challenged mice. In high-density regions the uptake was higher than that of regions of corresponding density in control animals. The oxygen delivery was proportional to the oxygen uptake in both groups.

**Conclusions:**

The proposed method allowed for the regional assessment of the relationship between lung density and two aspects of lung function, the oxygen delivery and uptake. When compared to global indices of lung function, an increased sensitivity for detecting heterogeneous lung disorders was found. This indicated that the technique has potential for early detection of lung dysfunction–before global changes occur.

## Introduction

Emphysema arising from the destruction of alveolar walls is one of the main conditions of chronic obstructive pulmonary disease (COPD)–presently the third leading cause of death in the world [1]. The lung-tissue breakdown leads to a loss of elastic recoil and to the enlargement of air spaces, causing airflow obstruction, impaired gas exchange, and lung hyperinflation [2].

In clinical trials, these conditions can be assessed using pulmonary function tests such as FEV1 (forced expiratory volume in 1 second), arterial blood gas analysis, and lung volume measurements at rest or during exercise [3]. However, their specificity and sensitivity as biomarkers of disease severity are limited, and investigation of other markers as alternative end-points is therefore of interest [3].

Several imaging biomarkers [4] for depicting lung disease heterogeneity have been proposed, allowing different disease phenotypes to be identified. For instance, Galban *et al*. [5] showed that computed tomography (CT) densitometry can distinguish different COPD phenotypes. The loss of parenchymal tissue (density) can also be detected by magnetic resonance imaging (MRI), being manifested as a reduction in proton density [6–9]. This technique has been applied for portraying the emphysema extent in COPD patients, where an agreement between MRI density mapping and CT densitometry was reported [8].

Besides characterizing lung parenchyma structural changes, various aspects of respiratory function can also be assessed via imaging modalities [10]. For example, oxygen-enhanced MRI (OE-MRI) has been proposed as a technique to assess lung function [11, 12]. Due to its paramagnetic nature, oxygen upon inhalation modulates the MRI signal by inducing changes in tissue relaxivity parameters. Shortening of T1 (the longitudinal relaxation time constant) is caused by oxygen dissolved in lung tissue and blood plasma, thereby reflecting oxygen uptake, whereas shortening of T2* (the transversal relaxation time constant) is caused by increased oxygen concentrations in the alveoli, providing information about the alveolar oxygen delivery [13, 14]. Hence, via the assessment of the two relaxation mechanisms (T1 and T2*), OE-MRI provides an opportunity for investigating the gas-exchange apparatus on a regional level.

In man, assessing oxygen uptake via OE-MRI has been explored in a range of pulmonary disorders over the last two decades. For instance, Ohno *et al*. [15] distinguished regions of low oxygenation in COPD patients with emphysema that correlated with a lower diffusing capacity for carbon monoxide. Since the amount of oxygen dissolved in the blood is modulated by the inhaled amount of oxygen, its diffusion through alveolar walls, and the perfusion of the surrounding capillaries, it has also been possible to cross-validate OE-MRI with other imaging techniques, such as ventilation assessments via hyperpolarized gases [16] and perfusion scanning [17], or lung morphology [18].

In the preclinical setting, the feasibility of performing OE-MRI in rodents has been demonstrated in naïve mice [19, 20], but few reports exist where OE-MRI has been used in models of pulmonary disease. Togao *et al*. [21] applied the technique to rats with pulmonary embolism, but no validation of OE-MRI in animal models of COPD has been presented.

Emphysema can be established in rodents via the instillation of porcine pancreatic elastase (PPE). The model is characterized by high reproducibility, and its cellular pathology and elastic properties have been extensively studied and validated against histopathological indices [22–25]. With regards to imaging studies, this model has been employed in work demonstrating that MRI can assess lung parenchyma destruction via the assessment of proton density [6, 7, 26, 27].

In the present work, the feasibility of employing MRI for the simultaneous assessment of both structural (lung density) and functional (oxygen delivery and oxygen uptake) information is demonstrated, with the hypothesis that the relationship between the parameters can be explored to gain increased sensitivity for investigating disease when assessed on a regional level. The work was performed in the mouse lung emphysema model described above, using a recently presented rodent OE-MRI imaging sequence [20].

## Materials and Methods

### Study Design

All experiments were performed in compliance with EU Directive 2010/63/EU. The protocol was approved by the local ethics committee (Göteborgs djurförsöksetisk nämnd, Permit Number: 263–2011). Female, eight-week-old C57BL6 mice were supplied from Taconic Europe, Denmark, with delivery body weights (BW) of 19–24 g. The mice were kept in an animal facility having a 12-hour light/dark cycle, at 60% relative humidity and a temperature of 22°C for one week before the study was initiated. Animals were fed a standard pellet diet (R70, Lantmännen, Sweden) and had access to tap water *ad libitum*.

Two groups of ten mice were included in the study to assess lung structure and function via MRI. In one group of animals, porcine pancreatic elastase (PPE, Sigma-Aldrich, Sweden, 6.5 U/mL, 50 μl/20 g BW) was instilled intranasally, whereas animals in the control group were instilled with saline (B. Braun Medical AB, Sweden, 0.9%, 50 μl/20 g BW). The instillation procedure was performed at day 0 and at day 7, followed by MRI examination at day 19 –a time-point when the presence of inflammatory fluids was expected to be low [26]. In separate cohorts blood gas analyses (ten PPE-challenged animals and eight control animals) and pulmonary functional tests (five PPE-challenged animals and five control animals) were performed, also at day 19 after the initial challenge.

### MRI Acquisition

The MRI experiments were performed on a Bruker BioSpec 47/40 4.7 T MR scanner (Bruker BioSpin GmbH, Germany) using a quadrature transmit/receive coil (diameter = 35 mm, Rapid Biomedical GmbH, Germany). The body temperature was maintained at 36.0 ± 0.2°C using heated circulating fluorocarbon, and monitored via a rectal probe (DM 852, Ellab A/S, Denmark). Anaesthesia (Isoflurane, Abbott Laboratories, USA, dose = 2.7%, flow = 340 ml/min) and carrier gases were delivered through a nose cone, allowing the animals to breath spontaneously (60–70 breaths per minute).

Single-slice image acquisition was performed with the animals in the supine position using a segmented inversion-recovery ultrashort echo-time (SIR-UTE) radial-encoding sequence with a global inversion pulse, as described previously [20]. The sequence parameters were: 400 radials/image, 3.4 ms intra-segment time, 0.5 ms echo time (TE), 10 segments, 40 projections per segment, 40 mm field-of-view, 1.6 mm slice thickness, 12° flip angle, and two averages. Seven inversion times (100, 400, 700, 1800, 3000, 4500 and 6000 ms) were used with an acquisition time of 2.1 minutes each. Images were acquired first while animals were breathing air and then while breathing 100% oxygen. The delay after switching gases was two minutes, in order for the gases to equilibrate. In total, the length of the protocol was approximately 30 minutes.

### Image Processing and Analysis

The images were reconstructed using an in-house algorithm written in IDL (Interactive Data Language, RSI 6.4, USA). A three-parameter fitting routine was used for pixel-level assessment of the longitudinal relaxation rate (R1 = 1/T1) and the signal at full relaxation (S0) as previously described [20]. The S0 of the air measurement (S0_air_) was used as an approximation of the relative lung proton density. ΔR1 (oxygen uptake) and ΔR2* (oxygen delivery) maps were generated via ΔR1 = R1_O2_–R1_air_ and ΔR2* = ln(S0_air_/S0_O2_)/TE. Regions of interests (ROIs) covering the full extent of the lung within the acquired slices were selected manually from TI = 6000 ms images, without excluding large blood vessels. The averages and the standard deviations within each ROI were calculated for S0_air_, R1_air_, R1_O2_, ΔR1, and ΔR2*.

The relationships between functional parameters (oxygen delivery, oxygen uptake) and lung structure (density) were investigated by grouping the lung pixels from all animals into twenty bins (with an equal number of approximately 1,000 pixels in each) based on their density values in order to reduce the influence of noise. Average R1_air_, R1_O2_, ΔR1, and ΔR2* were calculated for the pixels in each bin, allowing for the comparison of relaxivity-derived parameters in low and high-density locations. The relationship between ΔR1 and ΔR2* was investigated in the same way, with pixels being assigned to bins based on ΔR2* values.

### Histopathology

After imaging, the animals were euthanized with an overdose of pentobarbital sodium (250 mg/kg *i*.*p*.) and their lungs were collected for histopathological examination. Lungs were perfused through the interventricular septum with phosphate-buffered saline (Sigma Aldrich, Sweden) and then inflated with formaline (neutral buffered 10%, Sigma Aldrich, Sweden) via the trachea using gravity inflation at 25 cm H_2_O. The trachea was tied and the inflated lungs removed and placed in formalin for 24 hours before continued processing and embedding in paraffin. Two 3 μm sections, comprising both the left and the right lobes, were cut at the level of the tracheo-bronchial tree and stained with hematoxylin and eosin (H&E) before microscope examination.

### Blood-Gas Analysis

A blood-gas analysis was performed to assess the oxygenation status after the induced alveolar septa destruction. The same experimental setup was used as in the imaging study, except that the animals were not placed in the scanner and that the measurements were only performed with the animals breathing air. After breathing rate stabilization, a carotid was exposed surgically, and a blood volume of 100 μL was withdrawn. The arterial blood-gas characteristics, including blood pH, pCO_2_, pO_2_, HCO_3_^-^, Hgb, and O_2_ saturation, were assessed using a test cartridge blood analysis system (I-STAT System, USA).

### Pulmonary Function Test

A pulmonary function test was performed using the FlexiVent system (SCIREQ, Montreal, Canada). Mice were anaesthetized via an intraperitoneal injection of ketamine (100 mg/kg), xylazine (20 mg/kg), and acepromacine (3 mg/kg). An 18-gauge needle was used to cannulate the trachea and then connected to the FlexiVent. The animal was ventilated with a tidal volume of 10 ml/kg, at a frequency of 150 breaths per minute, and a positive end-expiratory pressure of 2.5 cmH_2_O. Two total lung capacity perturbations were performed to normalize the lung volume, followed by a snapshot perturbation and a pressure volume loop assessment with constant increasing pressure. Three snapshot perturbations with a 20 sec. delay between each were performed to assess the mean resistance and compliance of the whole lung. Following data collection, animals were removed from the ventilator and euthanized via terminal exsanguination.

### Statistical Analysis

Comparisons of global-level parameters between the control and PPE-challenged groups were performed using two-sample t-tests. A permutation test [28] was used to investigate group differences in the bin-analysis. In N = 10,000 iterations, the group labels (control, PPE-challenged) were randomly permuted among the animals, whereupon the group mean differences for each bin were calculated. The fraction of permutations where the mean difference deviated more from zero than what the difference observed in the study did gave an approximation of the p-value. Similarly, a bootstrap procedure [29] was carried out to estimate the SDs of the binned data. All statistical tests were two-tailed tests.

## Results

### MR Image Data

All animals were imaged successfully. Prior to analysis, the image quality was carefully inspected, and three animals (one control and two PPE-challenged) were removed from the analysis. These animals had visible lobular regions of increased proton density, exclusively on images acquired during oxygen exposure, presumably due to atelactasis.

#### Global MR image assessment

**Fig 1:**
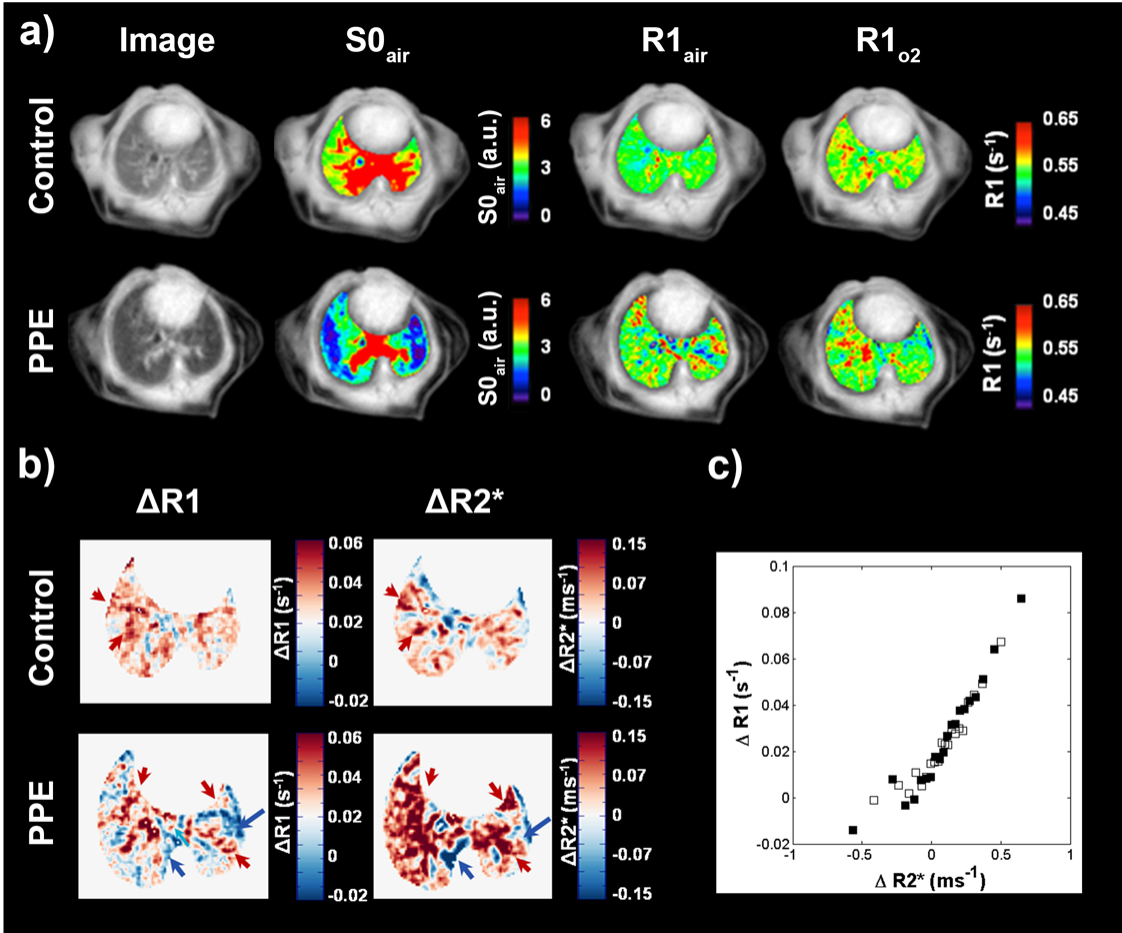
Representative TI = 6000 ms image, lung density (S0_air_), R1_air_, R1_O2_ maps of a control and PPE-challenged mouse lung. The maps are overlaid on TI = 6000 ms image acquired when animals were breathing air (a). ΔR1 (oxygen uptake), ΔR2* (oxygen delivery) maps for the same control and a PPE-challenged mouse (b). The challenged mouse exhibits less uniform distributions of ΔR1 and ΔR2* suggesting that the oxygen uptake and delivery to the lung were more heterogeneous. Regions of spatially matched ΔR1 and ΔR2* are indicated by arrows. Mean ΔR1 assessed in 20 bins based on ΔR2* for control (open squares) and PPE-challenged (filled squares) mice, showing excellent correlation between ΔR1 and ΔR2* (c).

The S0_air_, R1, ΔR1, and ΔR2* maps for PPE-challenged animals appeared more heterogeneous than maps for control animals, indicating a less uniform lung density, alveolar oxygen delivery, and oxygen uptake respectively (Fig 1A and 1B). Patterns present in 6000 ms TI images were often mirrored in the corresponding R1, ΔR1, and ΔR2* maps, and spatial variations present in ΔR1 and ΔR2* maps were generally matched (Fig 1A and 1B).

**Fig 2:**
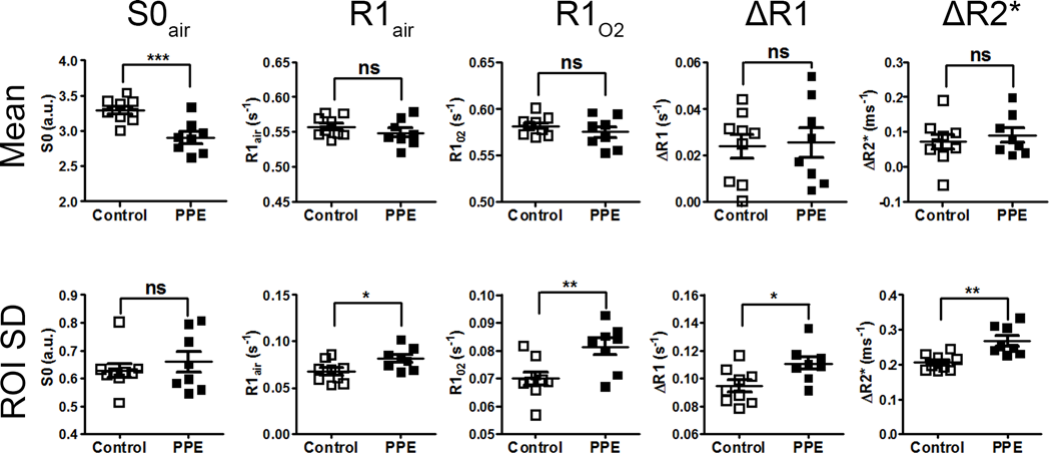
Global mean ± SEM values quantified from the whole lung ROI (top row) and the mean ± SEM standard variations within animals (bottom row) in control and PPE-challenged mice for density (S0_air_), R1_air_, R1_O2_, ΔR1, and ΔR2*. Although no group differences in global means were observed, besides in mean S0 _air_, the PPE-challenged group exhibited higher variances in R1_air_, R1_O2_, ΔR1, and ΔR2*. The p-values for statistical comparison (control *vs*. PPE) are denoted as follows: *p<0.05; **p<0.01; ***p<0.001.

Quantitative global MRI parameters are given in Table 1. The lung density (S0_air_) of the PPE-challenged group was 88.2 ± 8.8% (mean ± SD) of that of the control group (Fig 2A). Oxygen influence on R1 and R2* was detected in both groups. The R1 increases due to oxygen exposure (ΔR1) were similar in both groups (0.024 ± 0.015 s^-1^ control *vs*. 0.025 ± 0.018 s^-1^ PPE-challenged). Similarly, no difference in ΔR2* was found between groups (0.071 ± 0.065 ms^-1^ control *vs*. 0.090 ± 0.058 ms^-1^ PPE-challenged) (Table 1 and Fig 2).

The ROI standard deviations of R1_air_, R1_O2_, ΔR1, and ΔR2* were higher in the PPE-challenged group (Table 2), consistent with the heterogeneous distribution of these parameters observed in the maps.

**Table 1:** MRI-derived parameters for control and PPE-challenged animals (mean±SD). The p-values refer to two-sample t-tests of control and PPE-challenged animals.

	Mean control (n = 9)	Mean PPE (n = 8)	p-value
S0_air_ (a.u.)	3.29 ± 0.16	2.90 ± 0.23	0.001
R1_air_ (s^-1^)	0.557 ± 0.015	0.549 ± 0.019	0.35
R1_O2_ (s^-1^)	0.581 ± 0.010	0.574 ± 0.016	0.31
ΔR1 (s^-1^)	0.024 ± 0.015	0.025 ± 0.018	0.85
ΔR2* (ms^-1^)	0.071 ± 0.065	0.090 ± 0.058	0.54

**Table 2:** MRI-derived parameters for control and PPE-challenged animals (mean±SD). The p-values refer to two-sample t-tests of control and PPE-challenged animals.

	SD control (n = 9)	SD PPE (n = 8)	p-value
S0_air_ (a.u.)	0.62 ± 0.07	0.66 ± 0.10	0.50
R1_air_ (s^-1^)	0.067 ± 0.012	0.081 ± 0.012	0.02
R1_O2_ (s^-1^)	0.067 ± 0.007	0.081 ± 0.008	0.008
ΔR1 (s^-1^)	0.094 ± 0.012	0.110 ± 0.013	0.01
ΔR2* (ms^-1^)	0.205 ± 0.023	0.267 ± 0.041	0.001

#### Local MR image assessment

**Fig 3:**
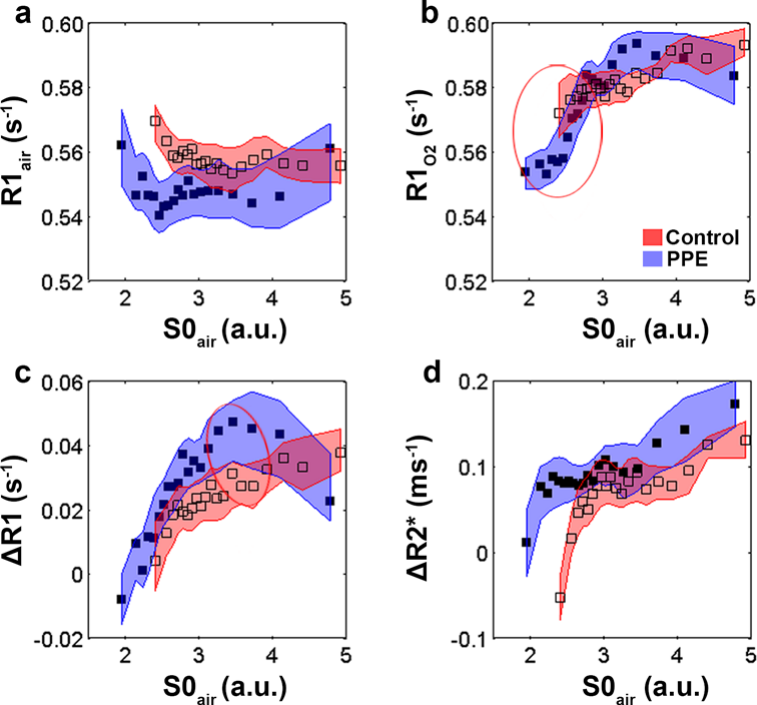
Mean ± SD assessed via a resampling procedure of R1air (a), R1O2 (b), ΔR1 (c), ΔR2* (d) determined in 20 bins based on density (S0air) for control (open squares), and PPE-challenged (filled squares) animals. Bins for which a test of equal mean showed p-values lower than 0.1 are indicated with a circle.

R1_air_ was fairly constant across all densities (Fig 3A) in both groups of animals. When animals were exposed to oxygen, a wider range of R1-values were present (Fig 3B), especially in the PPE-challenged group, where low-density regions exhibited lower R1 than what the high-density healthy regions did. In the ΔR1 data (Fig 3C), increased oxygen uptake was found at higher densities in both study groups, and again the PPE-challenged group covered a larger range. The lowest oxygen uptake across both groups was found in the lowest MR density regions of the PPE-challenged group, and interestingly, the highest oxygen uptake was found in high-density regions of the same group. No differences in alveolar oxygenation between the control and the PPE-challenged groups were observed in the ΔR2* data that could not be explained by the density shift between the two groups (Fig 3D). Again a tendency towards increased relaxivity values with increasing densities was found in both groups. Finally, strong correlations between ΔR1 and ΔR2* were detected both for control and for PPE-challenged animals (Fig 1), with no differences between the two groups.

### Histopathology

**Fig 4:**
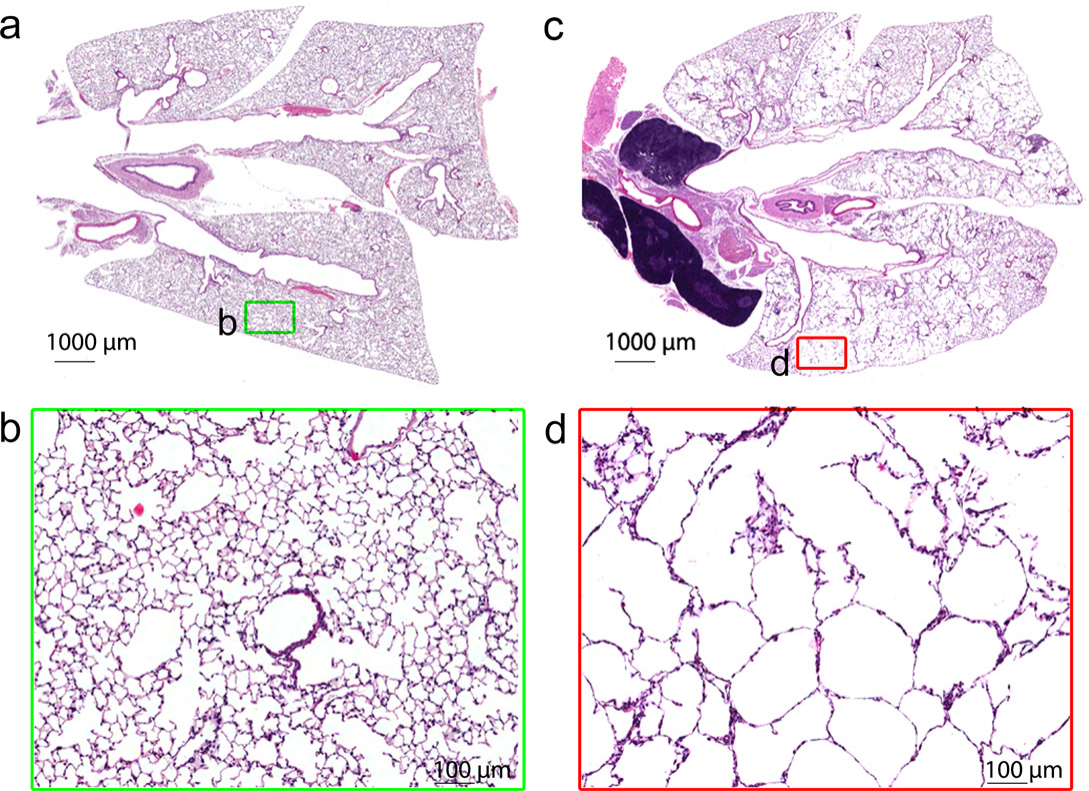
Low (a, c) and high magnification (b, d) sections of lung tissue for control (a, b) and PPE-challenged (c, d) mice. The squares indicate the magnified regions. Alveolar breakdown is present in parts of the section, whereas other parts resemble control tissue.

The histopathology evaluation revealed a patchy destruction of the inter-alveolar septa with moderate to severe loss of tissue connectivity in PPE-challenged animals (Fig 4). The enlargement of the alveolar spaces and destruction of the alveolar walls appeared multifocal in all lobes (Fig 4C). Evidence of perivascular lymphocytic inflammation was seen in all PPE-challenged animals, however the degree of inflammation was mild. No abnormalities were seen in the control mice.

### Blood-Gas Analysis

No detectable abnormalities in control and PPE-challenged animals were found in the measured blood-gas parameters (Table 3). Both groups exhibited blood-gas levels within the normal range.

**Table 3:** Blood-gas parameters for control and PPE-challenged animals (mean±SD).

	Control (n = 8)	PPE (n = 10)
pH	7.33 ± 0.04	7.35 ± 0.04
PaO_2_ (mm Hg)	122.5 ± 7.9	123.7 ± 6.7
PaCO_2_ (mm Hg)	41.0 ± 5.7	38.5 ± 3.7
[HCO_3_^−^] (mmol/L)	16.2 ± 1.4	16.4 ± 1.5
Hct (%PCV)	36.7 ± 2.1	37.5 ± 1.3
Hgb (10×g/L)	12.5 ± 0.7	12.6 ± 0.5
sO_2_ (%)	96.4 ± 0.7	97.0 ± 0.6

### Pulmonary Function Test

**Fig 5:**
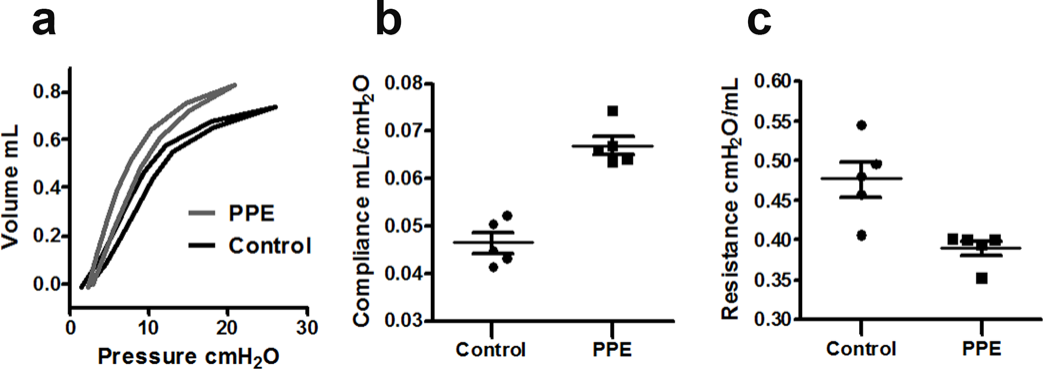
Pressure-volume loops for control and PPE-challenged mice assessed via FlexiVent. The curves represent group averages (a). Lung compliance (b) and resistance (c) in control and PPE-challenged mice.

Pressure-volume loop data for PPE-challenged mice demonstrated a left-upward shift corresponding to an increase of lung extensibility compared to controls (Fig 5A). The compliance of the lungs calculated from the FlexiVent snapshot perturbation was increased among PPE-challenged mice compared to controls (0.067 ± 0.002 mL/cmH_2_O *vs*. 0.047 ± 0.002 mL/cmH_2_O, p = 0.0001), while the resistance was reduced (0.39 ± 0.01 cmH_2_O/mL *vs*. 0.48 ± 0.02 cmH_2_O/mL, p = 0.01) Fig 5B and 5C.

## Discussion

In this study we demonstrated the feasibility of simultaneous mapping of lung density and function in a mouse model of PPE-induced emphysema. An ultrashort echo-time imaging sequence that enables the depiction of lung parenchyma destruction [6, 7] was combined with OE-MRI technique to assess the association between lung parenchyma density, oxygen delivery, and oxygen uptake. To the best of our knowledge this is the first OE-MRI study where the association between these three parameters is used to investigate how lung parenchyma destruction affects lung function (oxygen delivery, oxygen uptake) on a regional and global level in spatially matched images.

A reduction in lung parenchymal density as assessed via MRI was found in the PPE-challenged group, consistent with the performed histological evaluation. The same findings have previously been reported in PPE-challenged animals [6, 7, 26, 27], and can be attributed to a loss of alveolar walls and a partial destruction of the capillary bed, two features occurring in emphysema. The evidence of emphysema was also exhibited in functional assessments performed via FlexiVent, where an increased compliance and a left-upwards shift of the PV loops were found.

Despite the presence of a structural breakdown, no differences in global oxygenation (oxygen delivery and oxygen uptake) were found between the groups. The blood-gas analysis also confirmed that oxygen levels in PPE-challenged animals were normal. Although hypoxemia was reported previously in rats administered a similar dose of PPE per BW [30], the degree of emphysema in our model was not sufficient to cause global oxygenation changes. This result may be attributed to the presence of compensatory mechanisms preventing against hypoxemia [31].

When looking beyond the global analyses, notable differences between the two groups could be found in the MRI-derived metrics, demonstrating that the regional techniques had increased sensitivity for detecting pathological changes in the investigated model. The ROI standard deviations of R1_air_, R1_O2_, ΔR1, and ΔR2* were significantly higher in the PPE-challenged group than in the control group, indicating a more heterogeneous distribution of the alveolar oxygen concentration and the oxygen uptake. The analyses performed in different density intervals demonstrated dependencies between lung density and R1_O2_, ΔR1, and ΔR2*. In the low-density regions, lower values of R1_O2,_ ΔR1, and ΔR2* were seen, indicating a lower oxygen uptake in the lung parenchyma and lower gaseous oxygen levels in the alveoli, while the high-density regions revealed higher R1_O2_, ΔR1, and, ΔR2*, indicating the converse.

The lower ΔR1 enhancement in low-density regions of the PPE-challenged group may reflect a lower oxygen uptake due to the reduced oxygen diffusion that is associated with the destruction of capillaries in the emphysematous alveolar walls and the reduced surface area for gas exchange [15].

The regional analysis revealed a strong linear dependency between ΔR1 and ΔR2* (Fig 1), reflecting that in both groups, the amount of oxygen dissolved in the blood was proportional to the oxygen concentration in the alveoli. Hence, alternatively the reduced oxygen uptake in the damaged regions of PPE challenged animals could be a result of reduced regional ventilation. It is likely that the reduced elastic recoil in damaged regions of the lungs decreases the expiratory flow and compromises the respiratory mechanics [2].

The higher ΔR1 in the high-density regions of the PPE-challenged group suggests that the functional reserve of the lung has the ability to compensate for non-performing regions [31, 32]. To test this hypothesis, future studies could expose PPE-challenged animals to exercise stress [33].

ΔR1 as a metric assessing oxygen uptake has been reported in patients with a range of pulmonary disorders such as cystic fibrosis [34], emphysema [15], asthma [35], and COPD [36]. In agreement with our findings, patients exhibited distinct enhancement patterns. For instance, increased heterogeneity and the presence of low enhancement regions were reported earlier in COPD patients [37], although the relationship between functional parameters and lung structure was not investigated.

R1_air_ has been investigated previously as a marker of structural lung changes. For instance, Stadler *et al*. [38] proposed R1 increase to be a marker of human emphysema. Our results show that despite the presence of emphysematous lesions, no difference in R1 between the control and PPE-challenged groups could be observed. In addition, no R1 dependency on the density could be detected, further indicating that R1_air_ was not a sensitive marker of alveolar breakdown in this setting.

ΔR2* has previously been assessed via OE-MRI by first quantifying R2*_air_ and R2*_O2_ in separate experiments [13, 14]. As demonstrated here, this step is not necessary with the suggested protocol, since the expression ΔR2* = ln(S0_air_/S0_O2_)/TE holds. For the practical use of this technique the choice of TE is of importance, since if the TE is too short, the precision of ΔR2* estimates will be low due to the small signal difference between the two assessments. On the other hand, a too long TE may also be detrimental due to the overall reduction in signal.

The choice of TE also has implications for the density assessment (S0), since this relies on the assumption that TE is sufficiently short to prevent oxygen present in the air state from affecting the signal. In the present study, the global S_O2_-to-S_air_ ratio was on average 0.98 indicating that the approximation was valid, with the rationale being that the difference in signal between a hypothetical state without oxygen and the air state is expected to be smaller than the difference between the air state and the fully oxygenated state.

It has been shown previously that hybrid-imaging methods, for example single photon emission computed tomography (SPECT) combined with CT scanning improved diagnosis and subtyping of individuals with COPD [39]. Similarly, we postulate that the proposed method of simultaneous assessment of lung function and structure will improve the sensitivity for assessing lung pathology. The presented technique has potential value when assessing heterogeneous diseases such as COPD, where the ability to detect regional changes may be more meaningful than the detection of global changes. In addition, the technique provides an idea of the resolution scale at which disease can be detected, which may lead to a better understanding of the nature of disease progression and improved treatment management.

Although we consider these analyses promising for detecting regional differences within lung tissue, full lung parenchyma coverage via 3D or multi-slice approaches [40–42] should be considered in order to address heterogeneity issues within the total lung parenchyma. Increases in acquisition time could be avoided either at the expense of the spatial resolution or by applying advanced acquisition techniques such as compressed sensing [43].

Three separate animal cohorts were investigated in the study, and the respective results should be compared with some caution. Performing all tests on a single cohort was avoided, since the invasive nature of FlexiVent based on the forced oscillation technique, risked influencing the histological assessments, and the blood-gas analysis could be influenced by the air/oxygen breathing protocol.

In conclusion, we present an ultrashort echo-time imaging method enabling the quantification of alveolar oxygen concentrations and oxygen transported to the blood. Simultaneously, lung density maps demonstrating alveolar architecture damage are obtained, making it possible to investigate and exploit the relationships between functional and structural parameters on a regional level. In the investigated mouse PPE-induced emphysema model, a heterogeneous response to oxygen was found in the PPE-challenged group. Despite the fact that a global reduction in lung function was not present, regions with reduced lung function could still be identified by also taking the structural information into account. The combined imaging of spatially matched lung structure and function resulted in an increased detection sensitivity for tissue dysfunction, and provided additional insight into mechanisms of the underlying lung physiology.
